# Identification of hub genes in bladder cancer based on weighted gene co‐expression network analysis from TCGA database

**DOI:** 10.1002/cnr2.1557

**Published:** 2021-09-20

**Authors:** Lei Wang, Xudong Liu, Miao Yue, Zhe Liu, Yu Zhang, Ying Ma, Jia Luo, Wuling Li, Jiangshan Bai, Hongmei Yao, Yuxuan Chen, Xiaofeng Li, Dayun Feng, Xinqiang Song

**Affiliations:** ^1^ College of Life Sciences Xinyang Normal University Xinyang China; ^2^ College of Life Medicine Xinyang Normal University Xinyang China; ^3^ Department of Computer Science City University of Hong Kong Hong Kong China; ^4^ Department of Recovery Medicine People's Liberation Army 990 Hospital Xinyang China; ^5^ Department of Pathology First Affiliated Hospital of Xi'an Jiaotong University Xi'an China; ^6^ Department of Neurosurgery, Tangdu Hospital Fourth Military Medical University Xi'an China

**Keywords:** biomarker, MIBC, mutation, TCGA database, WGCNA

## Abstract

**Background:**

Muscular invasive bladder cancer (MIBC) is a common malignant tumor in the world. Because of their heterogeneity in prognosis and response to treatment, biomarkers that can predict survival or help make treatment decisions in patients with MIBC are essential for individualized treatment.

**Aim:**

We aimed to integrate bioinformatics research methods to identify a set of effective biomarkers capable of predicting, diagnosing, and treating MIBC. To provide a new theoretical basis for the diagnosis and treatment of bladder cancer.

**Methods and results:**

Gene expression profiles and clinical data of MIBC were obtained by downloading from the Cancer Genome Atlas database. A dataset of 129 MIBC cases and controls was included. 2084 up‐regulated genes and 2961 down‐regulated genes were identified by differentially expressed gene (DEG) analysis. Then, gene ontology analysis was performed to explore the biological functions of DEGs, respectively. The up‐regulated DEGs are mainly enriched in epidermal cell differentiation, mitotic nuclear division, and so forth. They are also involved in the cell cycle, p53 signaling pathway, PPAR signaling pathway, and so forth. The weighted gene co‐expression network analysis yielded five modules related to pathological stages and grading, of which blue and turquoise were the most relevant modules for MIBC. Next, Using Kaplan–Meier survival analysis to identify further hub genes, the screening criteria at *p* ≤ .05, we found *CNKSR1*, *HIP1R*, *CFL2*, *TPM1*, *CSRP1*, *SYNM*, *POPDC2*, *PJA2*, and *RBBP8NL* genes associated with the progression and prognosis of MIBC patients. Finally, immunohistochemistry experiments further confirmed that *CNKSR1* plays a vital role in the tumorigenic context of MIBC.

**Conclusion:**

The research suggests that *CNKSR1*, *POPDC2*, and *PJA2* may be novel biomarkers as therapeutic targets for MIBC, especially we used immunohistochemical further to validate *CNKSR1* as a therapeutic target for MIBC which may help to improve the prognosis for MIBC.

## 1 BACKGROUND

Muscular invasive bladder cancer (MIBC) is a highly heterogeneous cancer of the urinary system, and most bladder cancers are urothelial carcinomas. Currently, 25% of patients have muscle‐infiltrating or metastatic disease at the initial diagnosis and have a poor prognosis.^1^ Neoadjuvant cisplatin‐based chemotherapy (NAC) is the most effective approach and standard of care for MIBC before radical cystectomy.^2^ But many patients do not respond to NAC and patients with MIBC usually relapse within 2 years. A biomarker is a biological substance whose detection indicates a specific disease state.^3^ To date, several biomarkers have been introduced in daily clinical practice, including risk assessment, screening, differential diagnosis, prognostic determination, treatment response prediction, and disease progression monitoring.^4^

With the discovery and development of high‐throughput sequencing methods, the systematic analysis of high‐throughput sequencing data and screening of important information is the basis for subsequent studies.^5^ The emergence of network biology has led to a deeper understanding of complex biological systems, allowing the realization of tissue or cellular functions with a modular character. The development of cancer is a systems biology process (BP) that spans different functional networks.^6^ Weighted gene co‐expression network analysis (WGCNA)^7^ is a systems biology tool for characterizing gene expression patterns in samples and has been widely used in the analysis of various cancers,^8^ such as colorectal cancer,^9^ non‐small‐cell lung cancer (NSCLC),^10^ and breast cancer.^11^ WGCNA is used by studying the relationship between tissue microarray data and clinical features to identify possible biomarkers for predicting relevant cancers and comparing differentially expressed genes and studying the interactions between genes in different modules.^12^

In our study, the RNA sequencing (RNA‐seq) profile data of MIBC was downloaded from the Cancer Genome Atlas (TCGA) database. Then, the differentially expressed genes (DEGs) between MIBC and normal tissues were further analyzed at the expression and functional levels. After that, The gene ontology (GO) functional enrichment analyses of DEGs were performed by clusterprofiler R package. Subsequently, WGCNA was used to identify modules related to disease status, and pivotal genes for turquoise and blue modules were identified. Finally, the hub genes were verified by survival analysis, an independent dataset, and an immunohistochemical (IHC) experiment to determine these genes play an essential role in MIBC development. Therefore, our research may identify several effective biomarkers for MIBC and provide practical help for treating diseases.

## 2 MATERIALS AND METHODS

### 2.1 Data collection and processing

**FIGURE 1:**
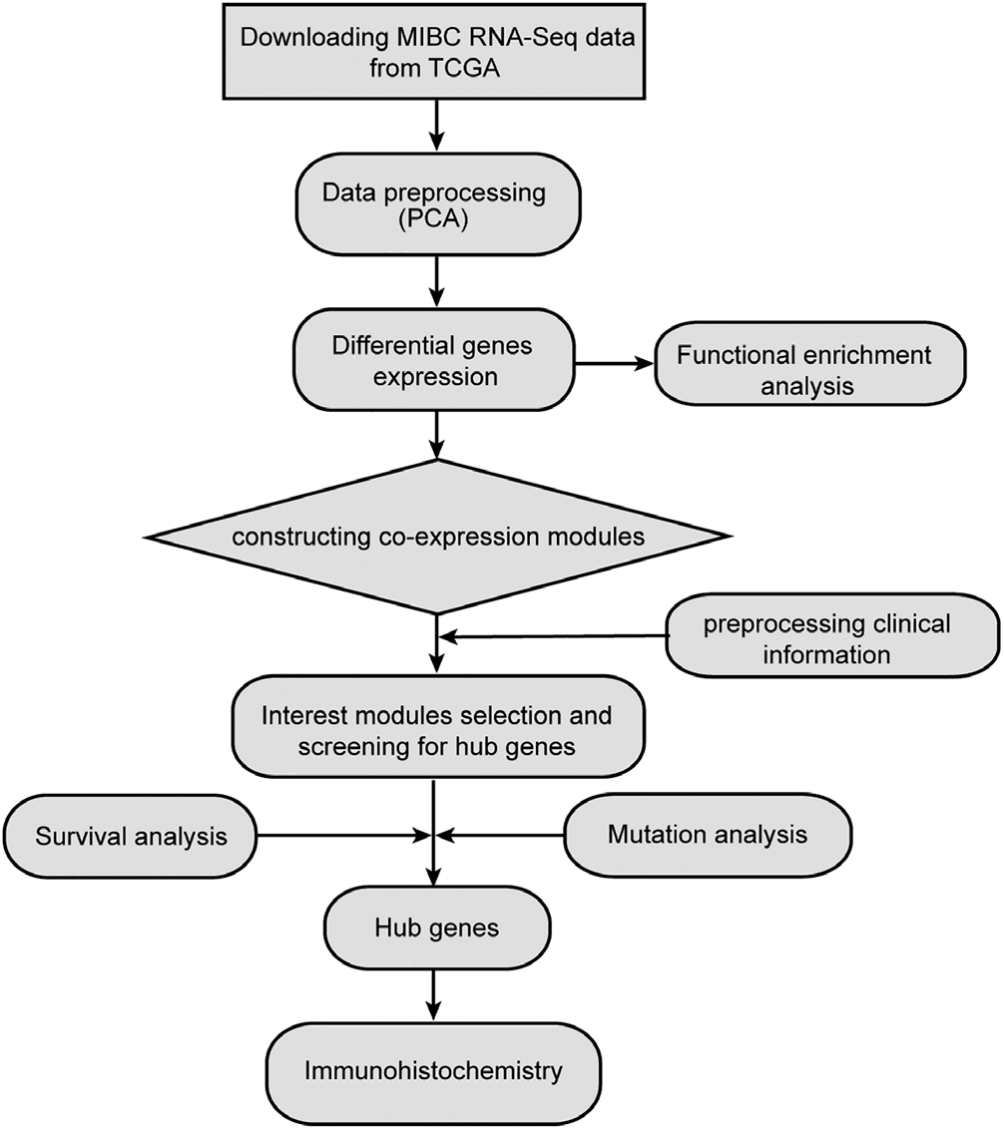
Flow chart of data collection, preparation, processing, analyzing, and validation in the study

The resource‐rich public database (TCGA: https://www.cancer.gov/tcga) provides insight into the mechanisms of cancer progression and the opportunity to discover new biomarkers. The RNA‐seq data included 19 normal and 414 tumor samples. Principal component analysis (PCA) was performed on the gene expression data of MIBC using the “factoextra” (version: 3.3.3) R package. A follow‐up analysis was performed after the exclusion of outliers. The workflow for our experimental study of the MIBC dataset in TCGA is shown in Figure 1.

### 2.2 Identification of differentially expressed genes

The “limma” R package (version: 3.3.3) was utilized to identify the DEGs between MIBC and normal samples in the dataset for validation. |Log2FC| > 2 and adj. *v*alue < .01 are used as cutoff criteria. We use the R package “ggplot2” (version: 3.3.3) to visualize the DEGs and show it with a volcano plot.

### 2.3 Gene ontology enrichment analyses of differentially expressed genes

A comprehensive understanding of the biological significance behind the genes is essential. GO is widely used for functional annotation and enrichment analysis; BP, molecular function (MF), and cellular component (CC) are the three major components of gene function.^13^ The Kyoto Encyclopedia of Genes and Genomes (KEGG) is a database resource that integrates genomic, chemical, and phylogenetic information. It enables efficient candidate genes for pathway enrichment analysis. In this study, GO enrichment analysis and KEGG pathway analysis of previously obtained DEGs were performed using the R package “clusterProfiler” (version: 3.14.3) and “org.Hs.eg.DB” (version: 3.10). *p* values < .05 for DEGs were considered statistically significant.

### 2.4 Co‐expression network construction

Weighted gene co‐expression networks analysis (WGCNA) of DEGs was performed according to the “WGCNA” (version: 1.70–3) R language package.^14^ WGCNA, which aims to find co‐expressed genes(modules) and explore the association between gene networks and phenotypes of interest and the hub genes in the network. Methodologically, WGCNA is divided into two parts: expression clustering analysis and phenotype association, which mainly include five steps: (1) network construction, (2) module identification, (3) relationship of modules and clinical traits, (4) topological property analysis, and (5) network visualization.

First, construct a scale‐free expression network degree, and used Pearson correlation matrix method and average linkage method for all two‐paired genes. Then, a weighted adjacency matrix was created using amn = |cmn|^β^ (cmn = Pearson's correlation between gene m and gene n; amn = adjacency between gene m and gene n).

$$a_{i}=\text{power}\;\left(s_{\mathrm{ij}},\beta \right)\;{\left|s_{\mathrm{ij}}\right|}^{\beta }.$$

*β* is a soft threshold parameter that emphasizes strong correlations between genes and penalizes weak correlations. After selecting the power of *β*, the neighborhood relationships are converted into a topological overlap matrix (TOM), which measures the network connectivity of a gene defined as the sum of its neighborhood relationships with all other genes for network generation corresponding similarity (1‐TOM) is calculated Average linkage hierarchical clustering was performed based on the TOM‐based dissimilarity measure to classify genes with similar expression profiles into gene modules. For the gene dendrogram, the minimum size gene group was 30. To further analyze the modules, we calculated the dissimilarity of module feature genes, selected a cut line for the module dendrogram, and merged some modules. Topological overlap measurements generated network modules with a power cutoff threshold of 3 and a module size cutoff of ≤50. The Pearson correlation test analyzed correlations between each module and clinical traits *p* < .05 was considered significant.

### 2.5 Identification of clinically significant modules

Two approaches were used to identify modules related to the clinical traits of MIBC. First, gene significance (GS) was defined as the log10 transformation of the *p* value (GS = lgP) in the linear regression between gene expression and the clinical traits. In addition, module significance (MS) was defined as the average GS for all the genes in a module. The module with the |MS| ranked first or second among all the selected modules was considered the one related to the clinical trait. Module eigengenes (MEs) were considered the major component in the principal component analysis for each gene module. The expression patterns of all genes could be summarized into a single characteristic expression profile within a given module. In addition, we calculated the correlation between MEs and clinical traits to identify the relevant module. The module with the maximal |MS| among all the selected modules was usually considered the one related to clinical traits. Finally, the module highly correlated with certain clinical traits was selected for further analysis.

### 2.6 Identification of hub genes

In this study, the key gene was defined by modular connectivity, measured by the absolute value of the module to measure the relationship between Pearson correlation (cor.gene Module Membership >0.8) and clinical traits, and measured by limiting the absolute value of Pearson correlation (cor.gene Trait Significance). Next, the Gene Expression Profiling Interactive Analysis (GEPIA) website (http://gepia2.cancer-pku.cn/) was used to verify the hub genes expression level, and the “survival” (version3.2–7) of R package was performed to Kaplan–Meier survival analysis to check hub genes were associated with prognostic significance.

### 2.7 Single nucleotide polymorphism validation of hub genes

The cBioportal (http://www.cbioportal.org/) database can provide a resource: visual analysis of multidimensional cancer genomic data. It also provides a graphical analysis at the gene level. We selected the bladder cancer database with 413 samples from cBioPortal to map the genome, including mutations, copy‐number variance (CNV), and mRNA expression z‐scores (RNASeqV2 RSEM). Meanwhile, we also showed the mutation types of some hub genes by the lollipop maps.

### 2.8 Immunohistochemical analysis

Clinical samples of MIBC were obtained from three MIBC patients from the Department of Pathology, Tangdu Hospital, Fourth Military Medical University. In preparation for using these clinical materials for research purposes, prior approval was obtained from the Patient Content and Institutional Research Ethics Committees. The IHC of the patient's MIBC tissue and its paired normal tissue sections is described previously.^15^ The *CNKSR1* antibody (product #10885‐1‐AP) was used to detect *CNKSR1* in this study. Two independent experts evaluated the results of the experiment. The scoring criteria of *CNKSR1* protein expression in MIBC samples are as follows: intensity score (− negative, + weak, + + moderate, + + strong) × positive reaction score (<10% −, 10% 25% +, 26% 50% positive +, > 50% moderate +).

### 2.9 Statistical analysis

The R software (version 3.6.1) was utilized for all statistical analyses of our study, and a *p* value <.05 was categorized as statistically significant.

## 3 RESULTS

### 3.1 Data collection and processing

**FIGURE 2:**
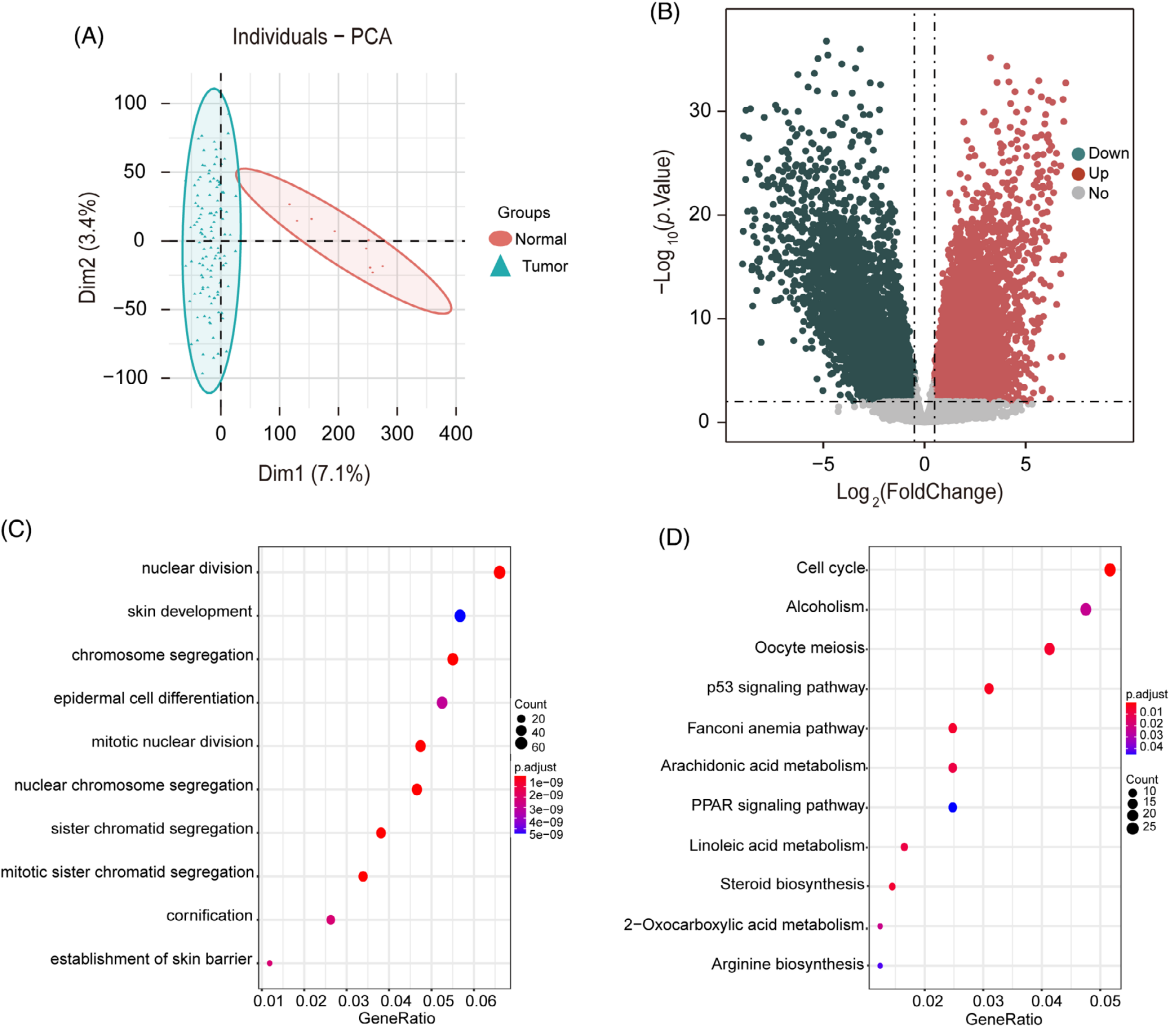
Differentially expressed genes ***analysis of transcription profile of muscular invasive bladder cancer (MIBC) and normal samples. (A) Principal component analysis showing the first 2 PCs of tumor and normal samples. (B) Volcano grams are used to show genes that are significantly differentially expressed in MIBC and normal samples. The red dots indicate the genes that are up‐regulated in MIBC samples, while the blue dots indicate the genes that are down‐regulated in MIBC samples. (C) Gene ontology on up‐regulated genes analyzed by difference analysis biological process. (D). KEGG on up‐regulated genes was analyzed by difference analysis

After principal component analysis, 120 tumor samples and nine normal samples were selected. The PCA result showed a significant difference between tumor samples and normal samples (Figure 2A). The Percentage of explained variances of the PC1 and PC2 of the data are 7.1% and 3.4%, respectively. The RNA‐seq data from these 120 samples were used in the subsequent studies.

### 3.2 Identification of differentially expressed genes and gene ontology functional annotation

A total of 2084 genes were up‐regulated, and 2961 genes were down‐regulated (Figure 2B). Then, the GO analysis results showed that the up‐regulated DEGs were mainly enriched in epidermal cell differentiation, mitotic nuclear division, and sister chromatid separation (Figure 2C). They are also involved in the cell cycle, p53 signaling pathway, PPAR signaling pathway, and son forth (Figure 2D). The results are consistent with the known dysfunctional process of MIBC, demonstrating the reliability of the method. The down‐regulated DEG enrichment results are as shown in Figure S1.

### 3.3 Identification of clinically significant modules

**FIGURE 3:**
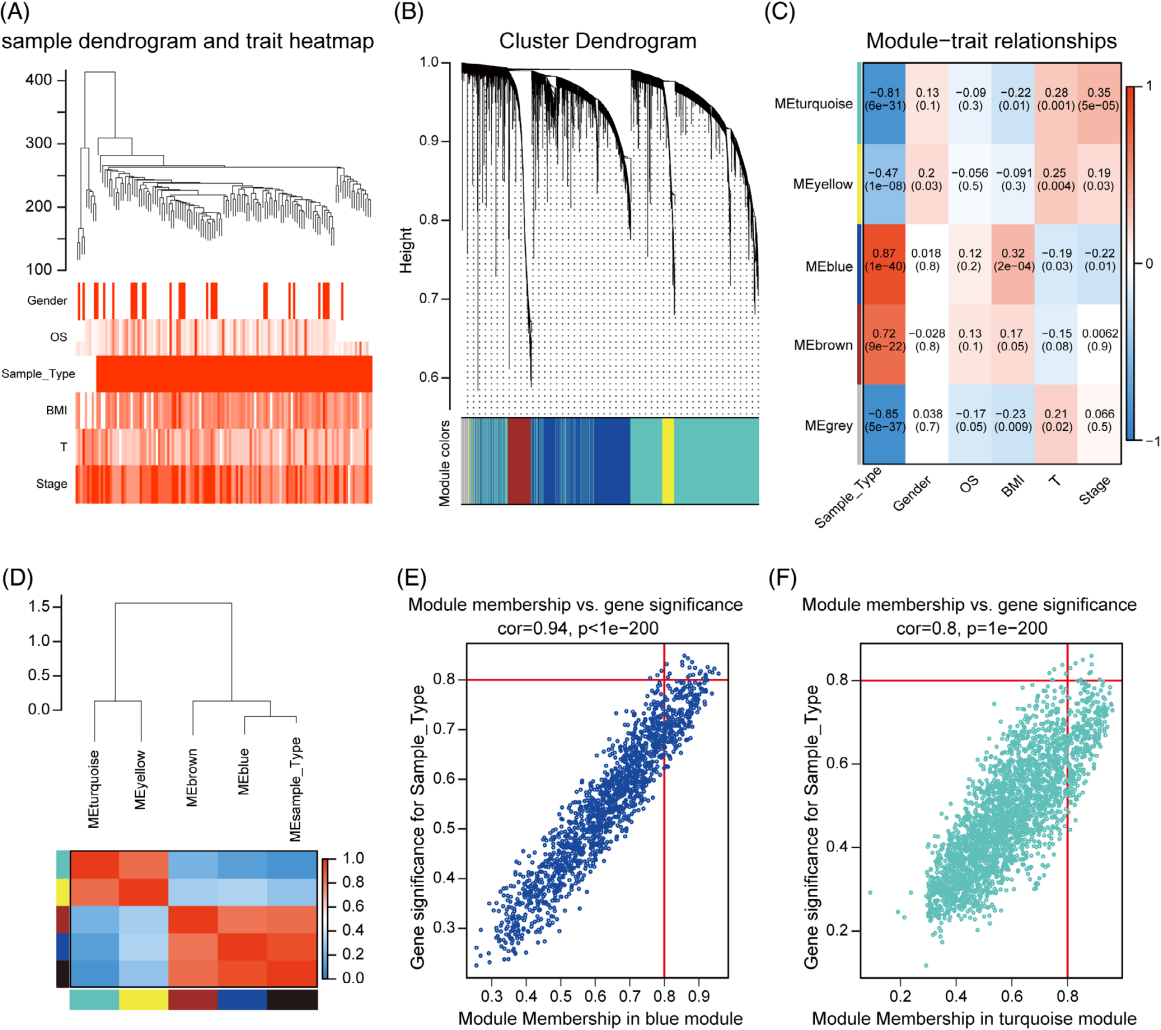
The identification of key modules related to muscular invasive bladder cancer tumorigenesis. (A) Clustering dendrograms of genes, with dissimilarity based on the topological overlap, together with assigned module colors. As a result, five co‐expression modules were constructed and were shown in different colors. (B) The eigengene dendrogram and heatmap identify groups of correlated eigengenes termed meta‐modules. (C) Module‐trait associations. (D,E) Scatter plot of module eigengenes in blue and brown module

The clinic traits data set was obtained from the TCGA database. To study the clinical significance of these modules, the correlations between MEs and clinical traits including Gender, OS, Tumor_Normal, BMI, T, and Stage were analyzed, evidencing that two modules were associated with the aforementioned clinical features by the *R*‐value of correlation, which is shown in Figure 3. The power of *β* = 7 was selected as the soft‐thresholding to ensure scale‐free networks (Figure S2). In addition, Module membership versus gene significance were blue module (cor = 0.94, *p* < 1e−200) and turquoise module (cor = 0.8, *p* = 1e−200), respectively (Figure 3E,F). This indicating that the above two modules apply to the Clinical Significance Module and allow further identification of hub genes associated with Sample_Type. The characteristic gene adjacency heat map is shown in Figure 3D, showing the correlation between adjacent blocks of modules. By analyzing the module relationship (MM) and gene significance (GS), we found higher values of MM in this module with GS (Figure 3E,F). Eleven genes were identified in the turquoise module, and 25 genes were identified in the blue module with thresholds of MM >0.8 and GS >0.8. Ultimately, these 36 genes were identified as relevant for tumor progression to analyze further and validate these hub candidate genes.

### 3.4 Survival and expression analysis of hub genes

**FIGURE 4:**
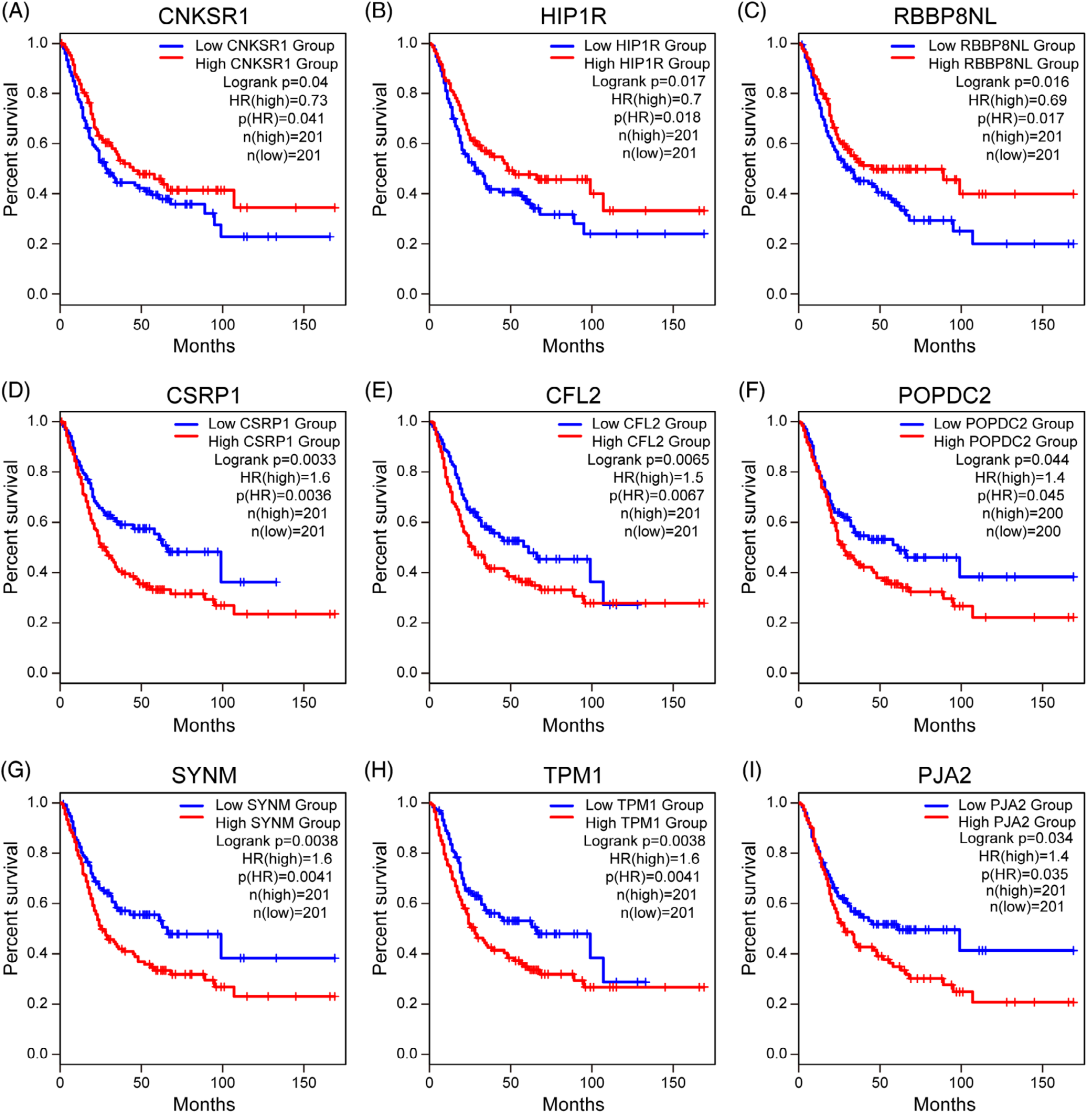
Survival analysis of the gene expression levels of nine hub genes using an independent dataset. (A–I) Expression levels of CNKSR1, HIP1R, RBBP8NL, CSRP1, POPDC2, CFL2, SYNM, TPM1, and PJA2 were significantly related to the overall survival of patients with MIBC (*p* < .05). Kaplan–Meier survival curves of muscular invasive bladder cancer cancer patients stratified by low or high expression of the nine hub genes

**FIGURE 5:**
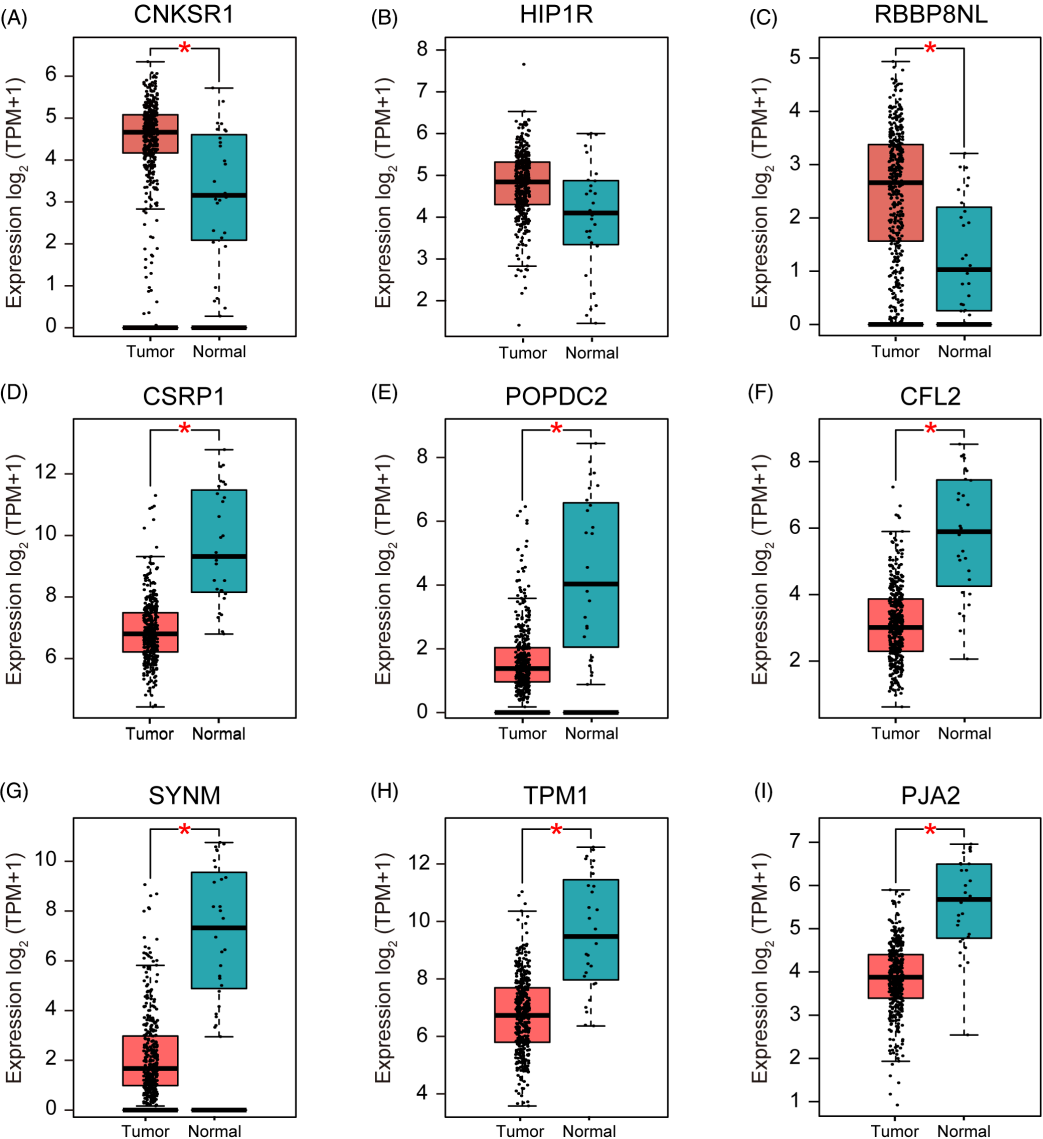
Validation of the gene expression levels of nine hub genes in muscular invasive bladder cancer (MIBC) (based on TCGA data in GEPIA). (A–I) Validation of the gene expression levels of PTTG1, RRM2, TOP2A, UHRF1, CEP55, BIRC5, UBE2C, FOXM1, and CDC20 are significantly upregulated in MIBC compared with normal tissues (*p* < .01). The red * represents *p* < .01

Gene expression validation was performed for all 36 genes, and the data were from the TCGA database through the GEPIA2 website (http://gepia2.cancer-pku.cn/) (Figure 4A,B). Since tumor progression always affects tumor prognosis, we investigated the role of these 36 genes in MIBC prognosis, including overall survival time. K‐M and log‐rank analysis showed that the important genes in the turquoise module were *CFL2*, *TPM1*, *CSRP1*, *SYNM*, *POPDC2*, and *CNKSR1*, *HIP1R*, *PJA2*, and *RBBP8NL* in the blue module (Figure 4A–I). Survival analysis using the GEPIA database was performed to estimate the relationship between the nine hub genes and the prognosis. As can be seen from the box‐profiles in Figure 5, the *CNKSR1*, *HIP1R*, and *RBBP8NL* were lower expressed in MIBC than normal bladder tissues (*p* < .05), and they may be tumor suppressor genes in MIBC. *CFL2*, *TPM1*, *CSRP1*, *SYNM*, *POPDC2*, and *PJA2* were highly expressed in normal tissues (Figure 5A–C). A previous study, which found differential expression of the *SYNM*, *TPM1*, *CSRP1* gene in bladder cancer, demonstrated the reliability of our study.^16^

### 3.5 Single nucleotide polymorphism analysis of hub genes

**FIGURE 6:**
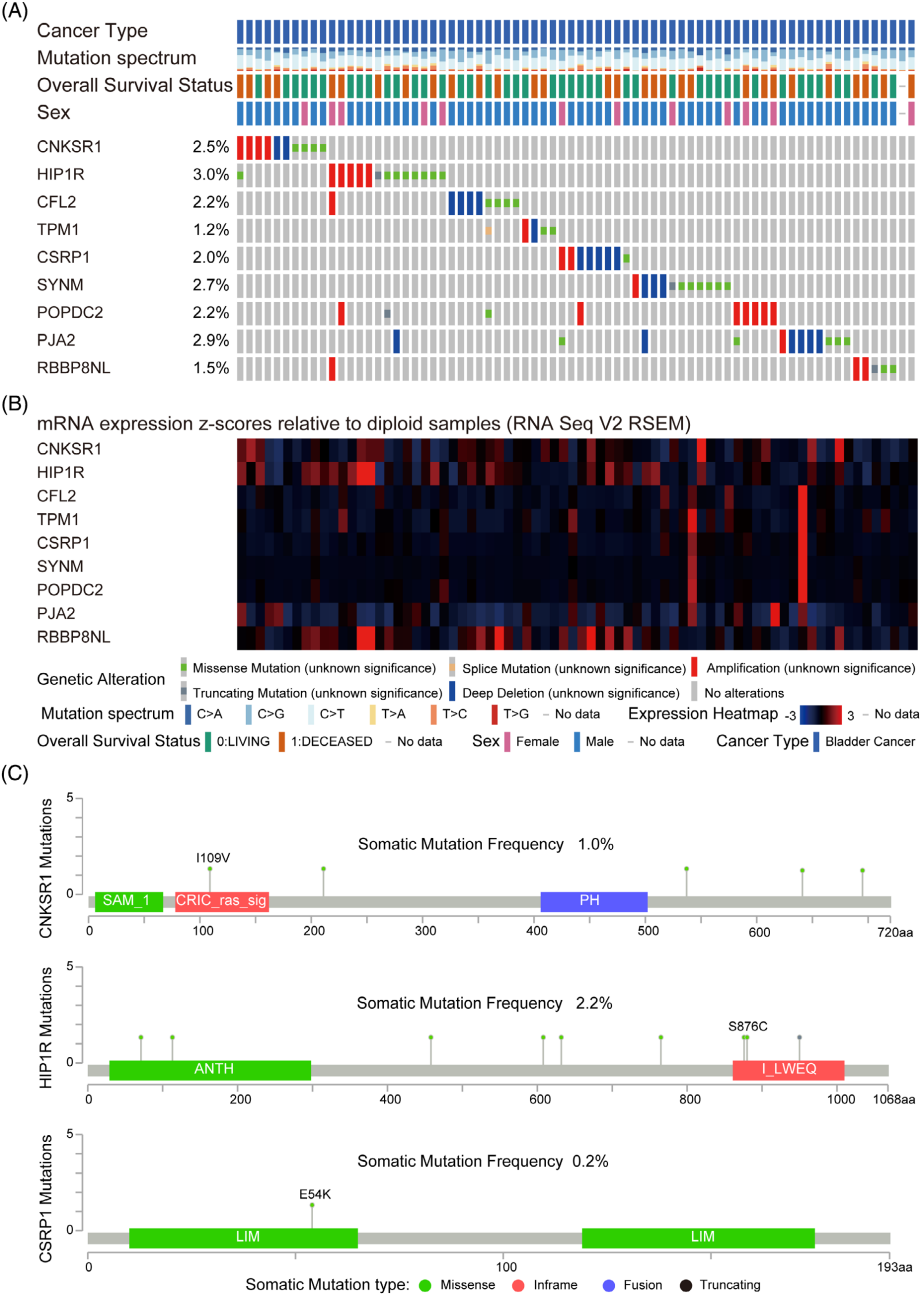
Genetic alterations associated with hub genes in muscular invasive bladder cancer (based on TCGA data in cBioPortal). Mutations nine hub genes based on TCGA data. Bar plots and heatmaps showing mutations in the nine hub genes. (B) Visualization of mutation types and mutation rates of single genes LIG1, CNKSRI, HIP1R, and CSRP1. (C) Lollipop plots showing the distribution of mutations in different domains of the proteins encoded by the nine hub genes

We used cBioPortal for Cancer Genomics (https://www.cbioportal.org) to verify the single nucleotide polymorphism of nine mutated hub genes and showed the mutation type and mutation rate of each gene (Figure 6A). Among them, the mutation rates of *RBBP8NL*, *HIP1R*, and *CNKSR1* were the highest, which were 12%, 7%, and 5%, respectively. And the site mutation type and mutation rate of *CNKSR1*, *HIP1R*, and other *CSRP1* in amino acid sequence (Figure 6B). The mutation rates and mutation types of the nine genes were shown in Table 1.

**TABLE 1:** Mutation types and mutation rates of genes

Gene	Somatic mutation frequency (%)	Somatic mutation types
CNKSR1	1.0	Missense
HIP1R	2.2	Missense Truncating
CFL2	1.0	Missense
TPM1	0.7	Missense Splice
CSRP1	0.2	Missense
SYNM	1.7	Missense Truncating
POPDC2	0.5	Missense Truncating
PJA2	1.2	Missense
RBBP8NL	0.7	Missense truncating

### 3.6 CNKSR1 immunohistochemical analysis

**FIGURE 7:**
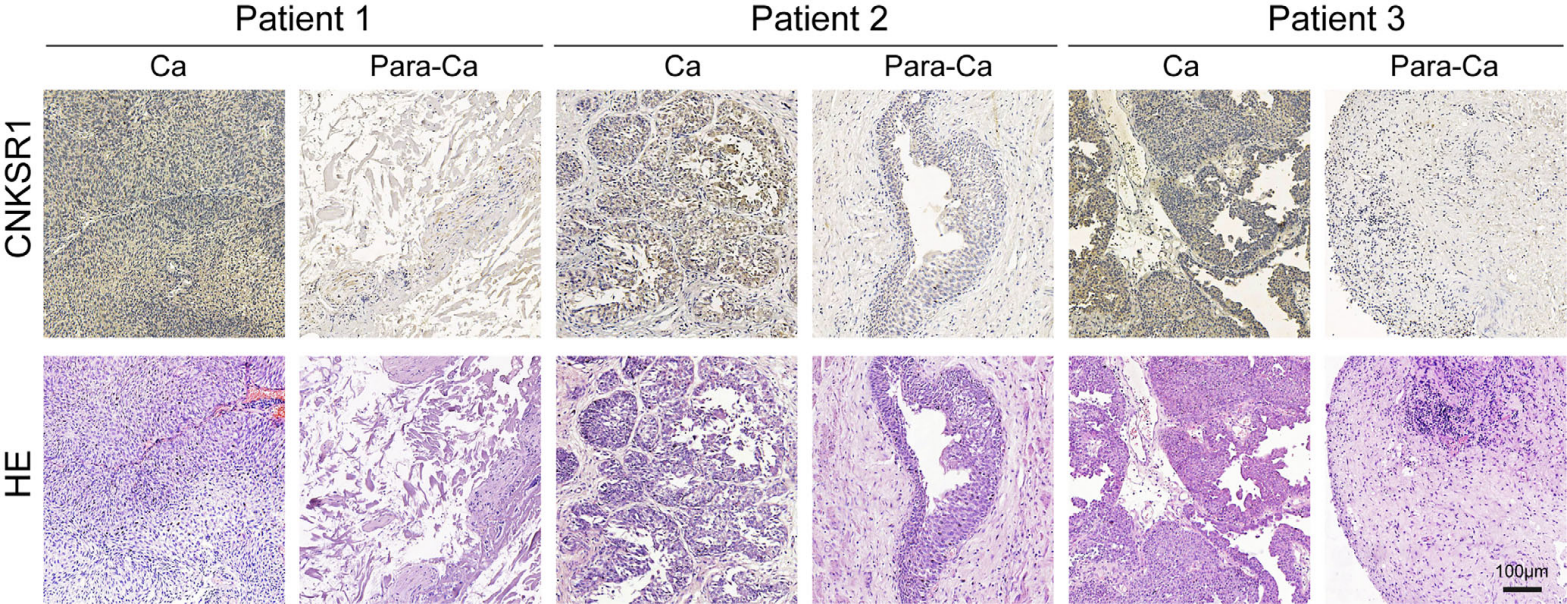
Immunohistochemistry CNKSR1 in tumor tissue (Ca) and adjacent normal tissue (Para‐Ca) from three patients with muscular invasive bladder cancer.Visualization of CNKSR1 genes in immunohistochemical analysis

As mentioned earlier,^17^ we used Proteintech‐branded *CNKSR1* antibody (10885‐1‐AP; Proteintech) for IHC analysis. Immunohistochemistry was analyzed by two independent researchers who were unaware of the clinical results. According to the Shimizu criteria standard,^18^ the expression of *CNKSR1* protein in MIBC samples ranged from 0 to 2+. *CNKSR1* was highly expressed in tumors as compared to matched normal, unaffected resected specimens (Figure 7). The expression levels of *CNKSR1* protein were divided into two low expression groups (0 or 1+) and one high expression group (2+). The experiments also suggested that the site of *CNKSR1* coloration was in the cytoplasm.

## 4 DISCUSSION

TCGA is a cancer research project established by the National Cancer Institute (NCI) in collaboration with the National Human Genome Research Institute (NHGRl) to provide an extensive, free reference database for cancer research by collecting and organizing a variety of cancer‐related histological data. The database covers genomic, transcriptomic, epigenomic, and proteomic data, providing a comprehensive, multidimensional data set. Despite significant improvements in the treatment of MIBC, it remains the most common malignancy with a high incidence in men worldwide.^19^ DNA microarray gene expression profile has been proved to have a specific application value and has been widely used to explore the differentially expressed genes involved in tumorigenesis and provide valuable information for clinical application.^20^ For MIBC patients with highly variable progression and prognosis, there is an urgent need for better and valuable biomarkers as prognostic or predictive molecules to provide patients with more useful clinical treatment strategies. In addition, these novel biomarkers could promote our understanding of tumorigenesis at the molecular level. Meanwhile, WGCNA as a method to screen indicators has many outstanding advantages over other methods, based on the association between co‐expression modules and clinic traits. Therefore, the WGCNA screening results have higher reliability and biological significance.

Clinical feature‐associated modules changed differently along with etiologies and histopathological characteristics, and two modules positively correlated with MIBC clinical traits were picked out. Function enrichment analyses for the turquoise module showed module hub genes involved in the TGF‐β signaling pathway, Wnt signaling pathway, and Ras signaling pathway. The reported study revealed that YY1 inhibits the EMT process in bladder cancer cells by reducing expression levels, regulating the TGF‐β pathway, and maybe a potential therapeutic target for future bladder cancer.^21^ Function enrichment analyses for the blue module showed module hub genes involved in the cAMP signaling pathway. cAMP is an important intracellular second messenger responsible for various cellular responses to external stimuli.^22^, ^23^ In previous studies, activation of the cAMP signaling pathway could be an important mechanism by which pro‐vascular propellants exert their therapeutic effects on lower urinary tract symptoms (LUTS).^24^ These findings could confirm our conclusions from another perspective. Meanwhile, according to previous studies, we found that *TPM1*,^25^ *SYNM*,^26^ *CSRP1*,^27^ *CFL2*,^28^ *HIP1R* ^29^ are often found in the cancer genetic sequence of bladder cancer.^16^, ^30^ The protein encoded by *TPM1* is a member of the widely distributed actin‐binding protein myosin (Tm) family, which participates in the contractile system of striated and smooth muscles and the cytoskeleton of non‐muscle cells, and studies have shown that *TPM1* is a tumor suppressor gene^31^ and plays a role in inhibiting the development of bladder urothelial carcinoma.^25^ It plays a key role in lymph node metastasis and may be a candidate marker of bladder cancer.^32^ Synemin (SYNM) is an IV‐type intermediate filament that has recently been shown to interact with the LIM domain protein zyxin, which may regulate cell adhesion and cell movement.^33^ For this diversity of potential functions associated with cancer development, studies have shown that *SYNM* genes are involved in carcinogenesis, such as, aberrant promoter methylation of the synemin gene is associated with early breast cancer recurrence,^26^ *SYNM* appears in pancreatic cancer,^34^ the oncogene sequence of hepatocellular carcinoma,^35^ and synemin expression in myofibromyopathy and other muscle diseases.^36^ The cofilin‐2 protein encoded by *CFL2* plays an important role in regulating sarcomere actin filaments. According to previous studies, the *CFL2* gene is a tumor suppressor gene in the oncogene sequence of bladder cancer, which has the biological significance of “Axon guidance”, “FC gamma R‐mediated”,^28^ and actin cytoskeleton regulation.^37^ DNA ligase I encoded by *LIG1* participates in DNA replication and repair. The function of *HIP1R* in the oncogene of bladder cancer is related to huntingtin interacting protein 1.^29^

The other three genes, *CNKSR1*, *POPDC2*, and *PJA2*, are also essential and highly involved in the process of many tumors. The *CNKSR1* gene encodes a connector enhancer for an enzyme, a kinase inhibitor of ras gene 1. This gene is an essential element in the receptor tyrosine kinase pathway and may be used to target tyrosine phosphorylation. It participates in the regulation of RAF in the MAPK pathway and may also play a role in the MAPK independent pathway. In addition, the PH domain of CNKSR1 combines with mut‐KRAS to inhibit the growth of mut‐KRA cells, which can treat a variety of cancers, such as pancreatic cancer.^38^ *POPDC2* belongs to the *POPDC* protein family. The *POPDC* protein is a promising target for anticancer therapy.^39^ The deletion of the *POPDC* gene and the inhibition of *POPDC* protein are related to the proliferation, migration, invasion, metastasis, drug resistance, and low survival ability of cancer cells in various human cancers. Overexpression of *POPDC* protein in vitro can inhibit the migration and invasion of cancer cells. For example, *POPDC* protein is used as a new target to inhibit the migration and proliferation of breast cancer cells.^39^ *POPDC2* was found to be present in cancer tissue sequences,^40^ such as prostate cancer.^41^ *PJA2* activates viral transcription by reducing the level of TCF/LEF1 by inhibiting Wnt/β‐catenin signal transduction, regulating KSR1 stability and mitotic signal,^42^ controlling PKA stability and signal transduction^43^ and *PJA2* ubiquitination of HIV‐1 Tat proteins with atypical chain bonds. We also found that the *PJA2* gene is often found in oncogene sequences of non‐small cell lung cancer,^44^ gastric cancer,^45^ lung cancer,^46^ glioblastoma,^47^ thyroid carcinoma,^48^ and so on.

To sum up, The research suggests that *CNKSR1*, *POPDC2*, and *PJA2* may be novel biomarkers as therapeutic targets for MIBC, especially we used IHC further to validate *CNKSR1* as a therapeutic target for MIBC which may help to improve the prognosis for MIBC.

## CONFLICT OF INTEREST

The authors declare that the research was conducted in the absence of any commercial or financial relationships that could be construed as a potential conflict of interest.

## ETHICAL STATEMENT

Not applicable.

## AUTHOR CONTRIBUTIONS

**Lei Wang:** Conceptualization (lead); data curation (lead); formal analysis (lead); funding acquisition (lead); investigation (lead); methodology (lead); project administration (lead); resources (lead); software (lead); supervision (lead); validation (lead); visualization (lead); writing – original draft (lead); writing – review and editing (lead). **Xudong Liu:** Conceptualization (equal); data curation (equal); formal analysis (equal); funding acquisition (equal); investigation (equal); methodology (equal); project administration (equal); resources (equal); validation (equal); visualization (lead); writing – original draft (lead); writing – review and editing (lead). **Miao Yue:** Conceptualization (equal); data curation (equal); formal analysis (equal); funding acquisition (equal); investigation (equal); methodology (equal); project administration (equal); software (equal); supervision (equal); validation (equal); visualization (lead); writing – original draft (lead); writing – review and editing (lead). **Zhe Liu:** Project administration (equal); resources (equal); software (equal); supervision (equal); validation (equal); visualization (equal); writing – review and editing (equal). **Yu Zhang:** Data curation (equal); formal analysis (equal); supervision (equal); validation (equal); visualization (equal); writing – review and editing (equal). **Ying Ma:** Data curation (equal); formal analysis (equal); supervision (equal); validation (equal); visualization (equal). **Jia Luo:** Software (equal); supervision (equal); validation (equal); visualization (equal). **Wuling Li:** Funding acquisition (equal); investigation (equal); methodology (equal); supervision (equal). **Jiangshan Bai:** Software (equal); supervision (equal); validation (equal); visualization (equal). **Hongmei Yao:** Resources (equal); software (equal); supervision (equal); validation (equal); visualization (equal). **Yuxuan Chen:** Software (equal); supervision (equal); visualization (equal). **Xiaofeng Li:** Data curation (equal); supervision (equal); validation (equal); visualization (equal). **Dayun Feng:** Conceptualization (lead); data curation (lead); investigation (lead); project administration (equal); resources (equal); validation (lead); visualization (lead).

## Supporting information


**Figure S1** Go and KEGG of down‐regulated genes. (A) GO on down‐regulated genes analyzed by difference analysis Biological Process. (B). KEGG on down‐regulated genes was analyzed by difference analysis.

**Figure S2** Analysis of the scale‐free fit index for various soft‐thresholding Powers. Analysis of the mean connectivity for various soft‐thresholding powers. Scale‐free fit index and mean connectivity were plotted as functions of the soft‐thresholding power.

## Data Availability

Publicly available datasets were analyzed in this study. This data can be found TCGA database (https://www.cancer.gov/).
