# Supplementary figures and images for: Identification of hub genes in bladder cancer based on weighted gene co‐expression network analysis from TCGA database
Source: Cancer Rep (Hoboken). 2021 Sep 20;5(9):e1557. doi: 10.1002/cnr2.1557 (PMC9458504; doi:10.1002/cnr2.1557)

A

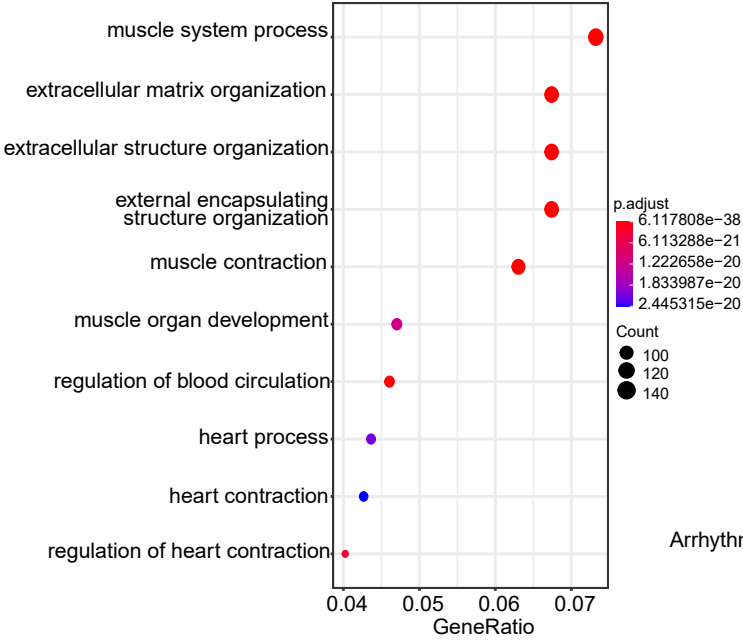

B

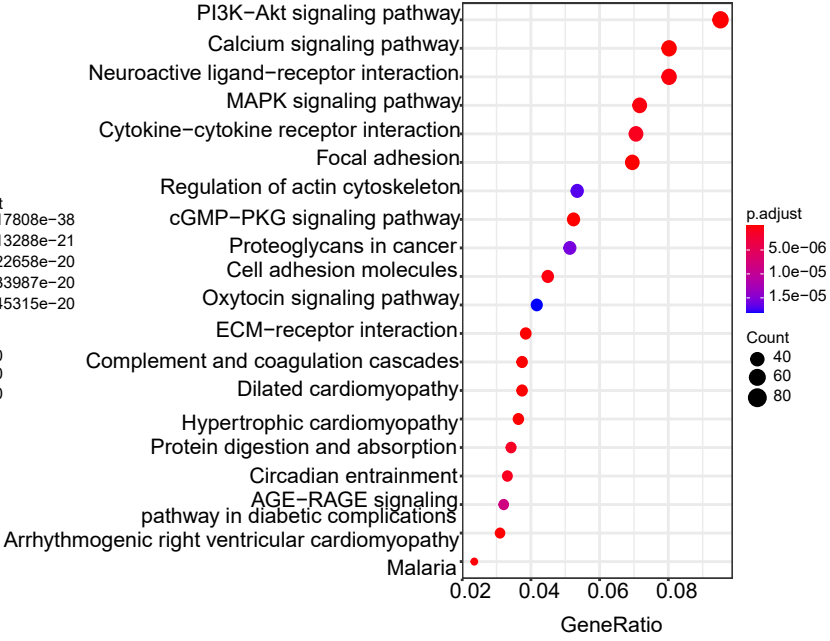

Supplement: Supplementary file 1 — Figure S1 Go and KEGG of down‐regulated genes. (A) GO on down‐regulated genes analyzed by difference analysis Biological Process. (B). KEGG on down‐regulated genes was analyzed by difference analysis. [file CNR2-5-e1557-s001.pdf]

Scale independence

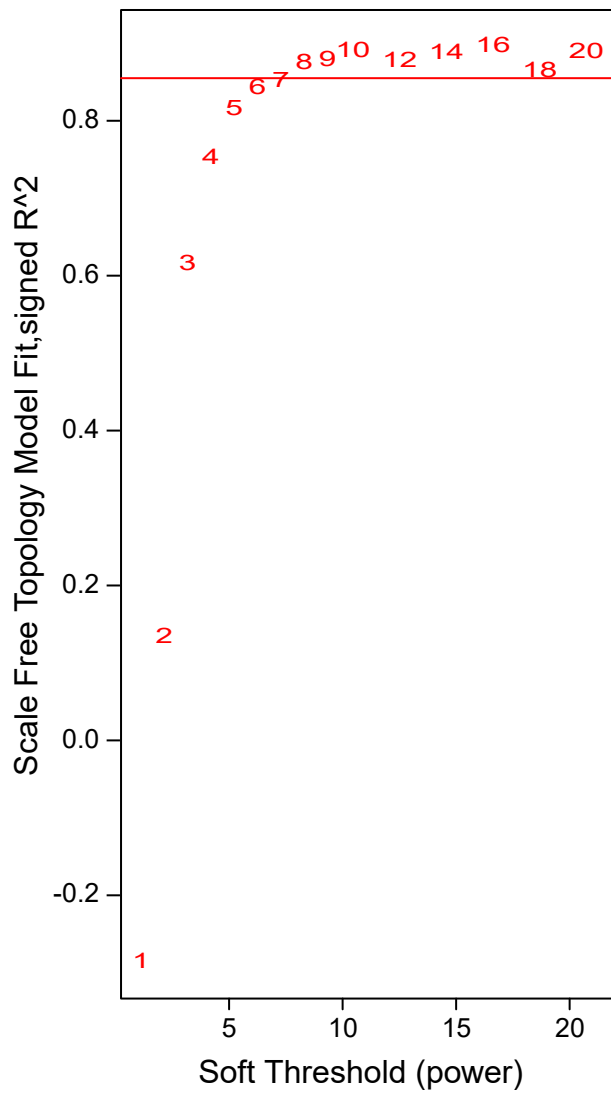

Mean connectivity

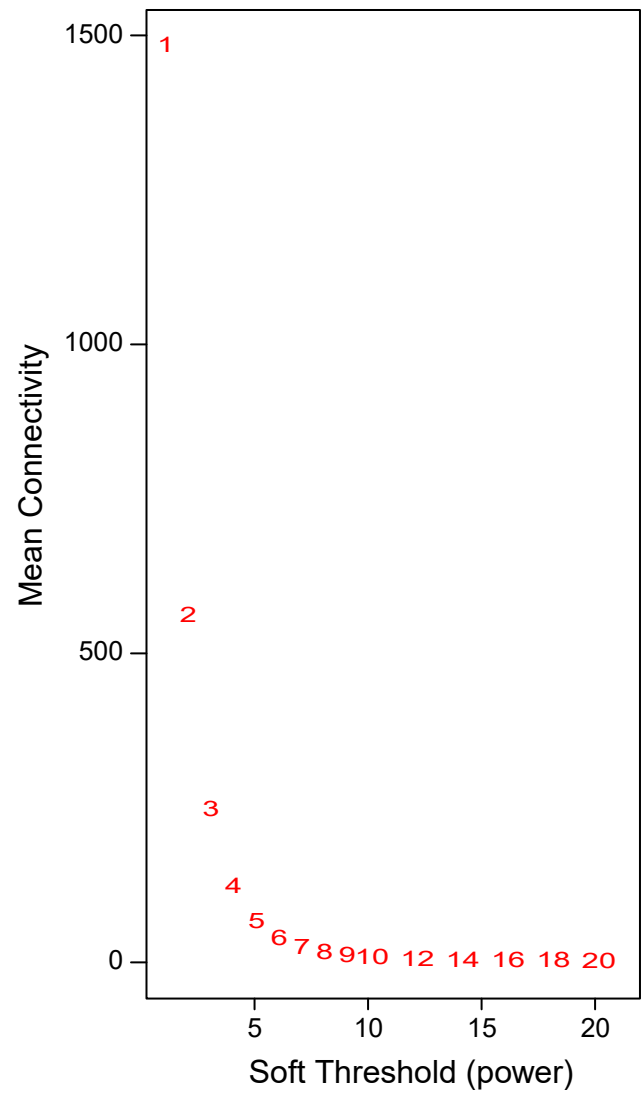

Supplement: Supplementary file 2 — Figure S2 Analysis of the scale‐free fit index for various soft‐thresholding Powers. Analysis of the mean connectivity for various soft‐thresholding powers. Scale‐free fit index and mean connectivity were plotted as functions of the soft‐thresholding power. [file CNR2-5-e1557-s002.pdf]
